# Case Report: Rare presentations of glioblastoma: three multifocal and one cerebellar cases highlighting the value of shared clinical experience

**DOI:** 10.3389/fonc.2026.1902596

**Published:** 2026-08-31

**Authors:** Nídia Maltez Cunha, Mónica Teotónio Fernandes, Clara Romero Sanchez, José Drago, Filipa Simões, José Ferreira, Daniel Bandarra, Joana Magalhães, Joaquim Pedro Correia, Paulo Luz

**Affiliations:** 1 Department of Medical Oncology, Unidade Local de Saúde do Algarve, Faro, Portugal; 2 Algarve Biomedical Center Research Institute (ABC-Ri), Faro, Portugal; 3 Algarve Biomedical Center (ABC), Faro, Portugal; 4 Faculdade de Medicina e Ciências Biomédicas (FMCB), Universidade do Algarve, Faro, Portugal; 5 Department of Neurosurgery, Unidade Local de Saúde do Algarve, Faro, Portugal; 6 Department of Neuroradiology, Unidade Local de Saúde do Algarve, Faro, Portugal

**Keywords:** brain tumors, case series, cerebellar glioblastoma, glioblastoma, multifocal glioblastoma, rare presentation

## Abstract

**Background:**

Glioblastoma (GBM) is the most common and aggressive primary brain tumor in adults, typically presenting as a single supratentorial lesion. Multifocal/multicentric, and infratentorial variants are rare but clinically significant, often mimicking metastatic disease and limiting the feasibility of standard treatment. Their management remains challenging, and dedicated therapeutic guidelines are lacking.

**Methods:**

We describe four uncommon GBM presentations, three multifocal supratentorial tumors and one cerebellar tumor, and integrate their clinical trajectories with a review of the literature to highlight diagnostic and therapeutic considerations.

**Results:**

Across cases, diagnosis required comprehensive neuroimaging and, in several instances, exclusion of an extracranial primary malignancy. Molecular profiles were consistent with *IDH*-wildtype GBM. Treatment feasibility varied substantially: one patient was unable to receive tumor-directed therapy because of poor performance status, one discontinued treatment after severe hematologic toxicity, one younger patient with preserved performance status initiated standard chemoradiotherapy, and the cerebellar GBM patient was not eligible for adjuvant therapy because of postoperative deterioration and posterior fossa involvement. Survival was short in three cases, ranging from approximately 50 days to 7.5 months after diagnosis, and was approximately one month in the cerebellar case.

**Conclusion:**

Rare presentations of GBM, including multifocal and cerebellar disease, carry substantial diagnostic ambiguity, limited therapeutic options, and a markedly poor prognosis. Early multidisciplinary evaluation, optimized supportive care, and research focused on tailored treatment strategies are urgently needed to improve outcomes in these challenging GBM subtypes.

## 1 Introduction

Glioblastoma (GBM) is a grade 4 glioma, a cancer that originates in glial cells or their precursors in the Central Nervous System (CNS). According to the World Health Organization (WHO) most recent guidelines, it should be diagnosed in the setting of isocitrate dehydrogenase (*IDH*)1/2-wildtype diffuse astrocytic glioma in adults if there is at least one of the features: microvascular proliferation, necrosis, *TERT* promoter mutation, *EGFR* gene amplification, and +7/-10 chromosome copy number alterations (1). Importantly, although it is a relatively rare cancer, it is the most prevalent and aggressive form of primary brain tumor in adults (2).

GBM usually presents as a unifocal, or single-lesion tumor characterized by rapid growth and diffuse infiltration into the surrounding brain tissue. Nevertheless, GBM may manifest as two or more distinct lesions within the brain, the so-called multifocal or multicentric GBMs, which are associated with a markedly poorer prognosis (3–6). Moreover, GBM is commonly localized in the supratentorial compartment, and locations like the brainstem and cerebellum are very rare (7).

For most patients with newly diagnosed GBM, the current standard of care is maximal safe surgical resection followed by radiotherapy with concomitant daily temozolomide (TMZ) and subsequent adjuvant TMZ, commonly referred to as the Stupp protocol, based on evidence demonstrating improved overall survival compared to radiotherapy alone (8). Despite a wide body of studies and clinical trials, contemporary guidelines and reviews confirm this multimodal approach established in 2005 as the backbone of treatment in fit adults, with adaptations in selected elderly patients. Alternatively, when the expected benefit of oncologic therapy is minimal, best supportive care with early integration of specialist palliative care is advocated (9–11).

Despite advancements in neuroimaging and, to a lesser extent, therapeutic approaches, multiple GBM remains a therapeutic challenge (12, 13). The complexity arises from several factors, including its heterogeneous nature and the difficulty in achieving maximal or complete surgical resection without compromising neurological function. Similarly to single/unifocal GBM, current standard treatment includes a combination of surgical resection, radiotherapy, and chemotherapy, but the outcomes are often suboptimal due to the multifaceted nature of the disease (3, 5, 6, 8, 14). Moreover, other less common presentations that include cerebellar GBM also pose challenges since their posterior fossa location limits safe resection, complicates radiotherapy planning, and often leads to rapid neurological deterioration, which reduces eligibility for standard chemoradiation (15, 16). Due to the higher complexity and worse prognosis, less common presentations of GBM have limited existing knowledge and no standardized treatment.

This case series aims to describe the clinical, radiological, histopathological, molecular, therapeutic, and outcome characteristics of four patients with rare GBM presentations observed at our institution, including three multifocal supratentorial GBMs and one cerebellar GBM. By reporting these cases together, we highlight how anatomically distinct uncommon GBM presentations may converge on similar clinical challenges, including diagnostic ambiguity, limited feasibility of maximal safe resection, difficulty completing standard chemoradiotherapy, rapid neurological decline, and the need for individualized multidisciplinary decision-making. Therefore, the objective of this study is to integrate our clinical experience with the available literature to identify practical lessons that may inform the management of these rare and aggressive GBM variants.

## 2 Case presentations

### 2.1 Case 1

**Figure 1:**
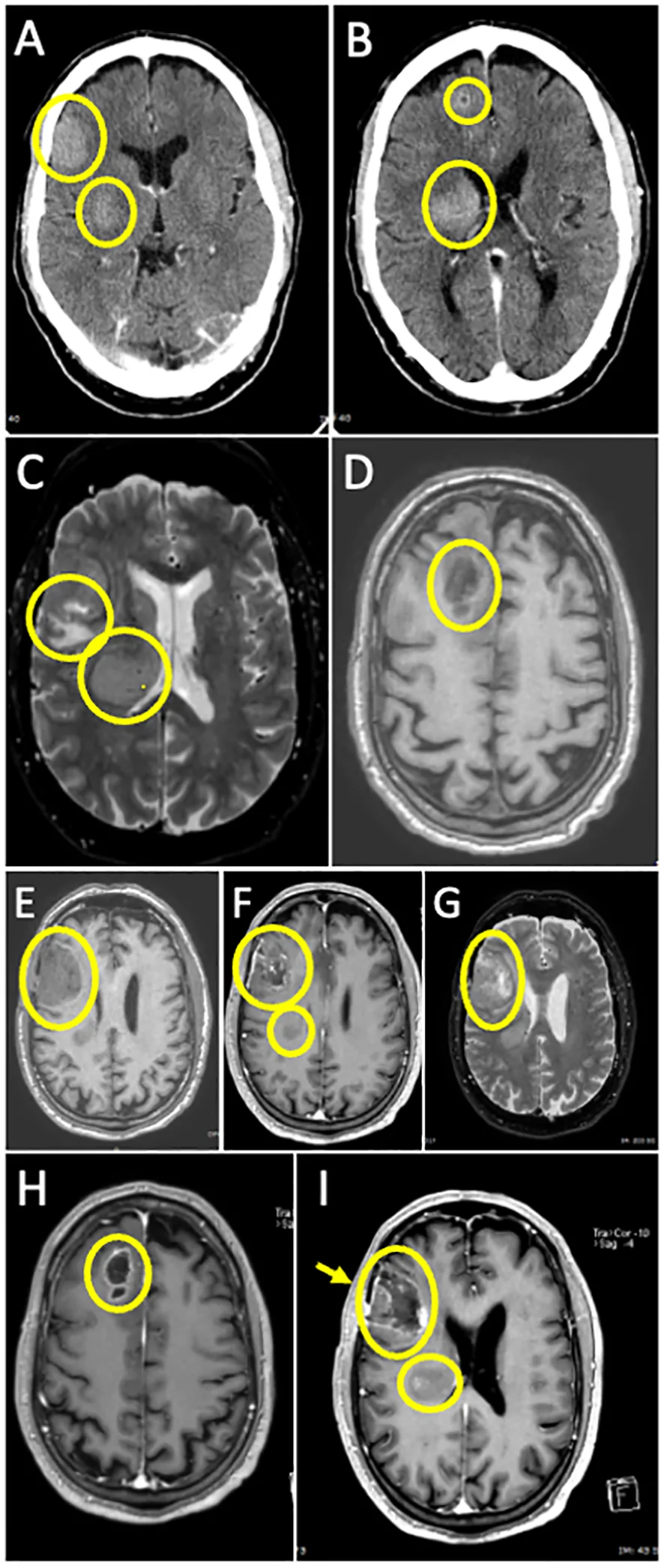
Preoperative computed tomography (CT) scans **(A, B)** and magnetic resonance imaging (MRI) **(C-H)** demonstrating multiple intra-axial lesions within the right cerebral hemisphere, consistent with multifocal disease. Yellow circles indicate the lesions. The largest lesion was located in the right frontal cortex, measuring 28 × 18 mm **(E-G)**. An additional lesion measuring 25 × 23 mm was identified in the deep periventricular region with involvement of the right thalamus **(C)**. Three smaller intra-axial nodules, each measuring approximately 10 mm, were also identified in the right hemisphere **(D, H)**. Postoperative MRI **(I)** shows the resection cavity, with the yellow arrow indicating the site of the resected right frontal lesion.

A 70-year-old Caucasian man, independent in all activities of his daily living, began experiencing complaints of paresthesia in his left upper limb and a sensation of weakness in the proximal regions of his lower limbs. He also reported a weight loss of 5 kilograms over the last 3 months. Initial cranial computed tomography (CT) revealed five space-occupying lesions suggestive of secondary lesions (**Figures 1A, B**). He was a former smoker, having quit more than 30 years before, with no significant personal medical history. Due to the onset of neurological deficits and suspected neoplasia with possible brain metastases from a primary tumor, the patient was admitted for further investigation of suspected brain metastases.

The magnetic resonance imaging (MRI) revealed five lesions, with the largest being a 28 x 18 mm lesion in the right frontal cortex (**Figures 1C, E-G**). Another 25 x 23 mm nodule was located in the deep periventricular area, also involving the right thalamus (**Figures 1C, I**). Three other small intra-axial micronodules, each approximately 10 mm in size, were identified in the right hemisphere (**Figures 1D, H**). No defined space-occupying lesions were found in the left cerebral hemisphere or infratentorial region, and no lytic lesions were observed on the bone plane. Extensive systemic evaluation, including whole-body imaging and targeted investigations, did not identify an extracranial primary malignancy.

Based on the presence of multiple right-hemispheric lesions without evidence of extracranial malignancy, this case was considered most consistent with multifocal GBM. A definitive multicentric classification was not assigned because the available imaging did not allow complete exclusion of microscopic anatomical continuity between lesions.

A brain biopsy was performed on the most accessible lesion with the removal of the right frontal lesion, through a right frontotemporal linear incision (**Figures 1I**, arrow). Histological analysis demonstrated fragments of an infiltrative glial neoplasm, hypercellular, with predominantly astrocytic morphology, slight pleomorphism, frequent mitoses, and microvascular proliferation. Immunohistochemical studies showed GFAP positivity, ATRX preservation, p53 negativity, a proliferative index (Ki-67/MIB-1) of 10-20%, and non-mutated *IDH*. The diagnosis was concluded as GBM.

Following this diagnosis, the patient was referred to a multidisciplinary neuro-oncology decision team, where the Stupp protocol was proposed and scheduled.

The patient tolerated treatment well during the initial weeks of concurrent chemoradiotherapy, remaining largely autonomous and living at home with his family. However, during the concomitant phase, he developed grade 4 neutropenia and grade 2 thrombocytopenia (Common Terminology Criteria for Adverse Events, CTCAE v5.0), resulting in a temporary interruption of treatment. A treatment-free interval of four weeks was required to allow for hematologic recovery. Following recovery, adjuvant TMZ was initiated and completed for one cycle (28 days).

Subsequently, the patient developed altered consciousness and a convulsive episode, accompanied by global clinical deterioration. Considering this progression, TMZ was discontinued, and management was shifted to exclusive best supportive care. The patient died two weeks later. Overall survival, calculated from histopathological diagnosis to death, was approximately 7.5 months.

### 2.2 Case 2

A 64-year-old male with previously identified multiple lesions but without a formal diagnosis was admitted to our institution because of progressive neurological deterioration. He presented with confusion, recent memory impairment, incoherent speech, gait instability, sphincter dysfunction, and intermittent psychomotor agitation.

**Figure 2:**
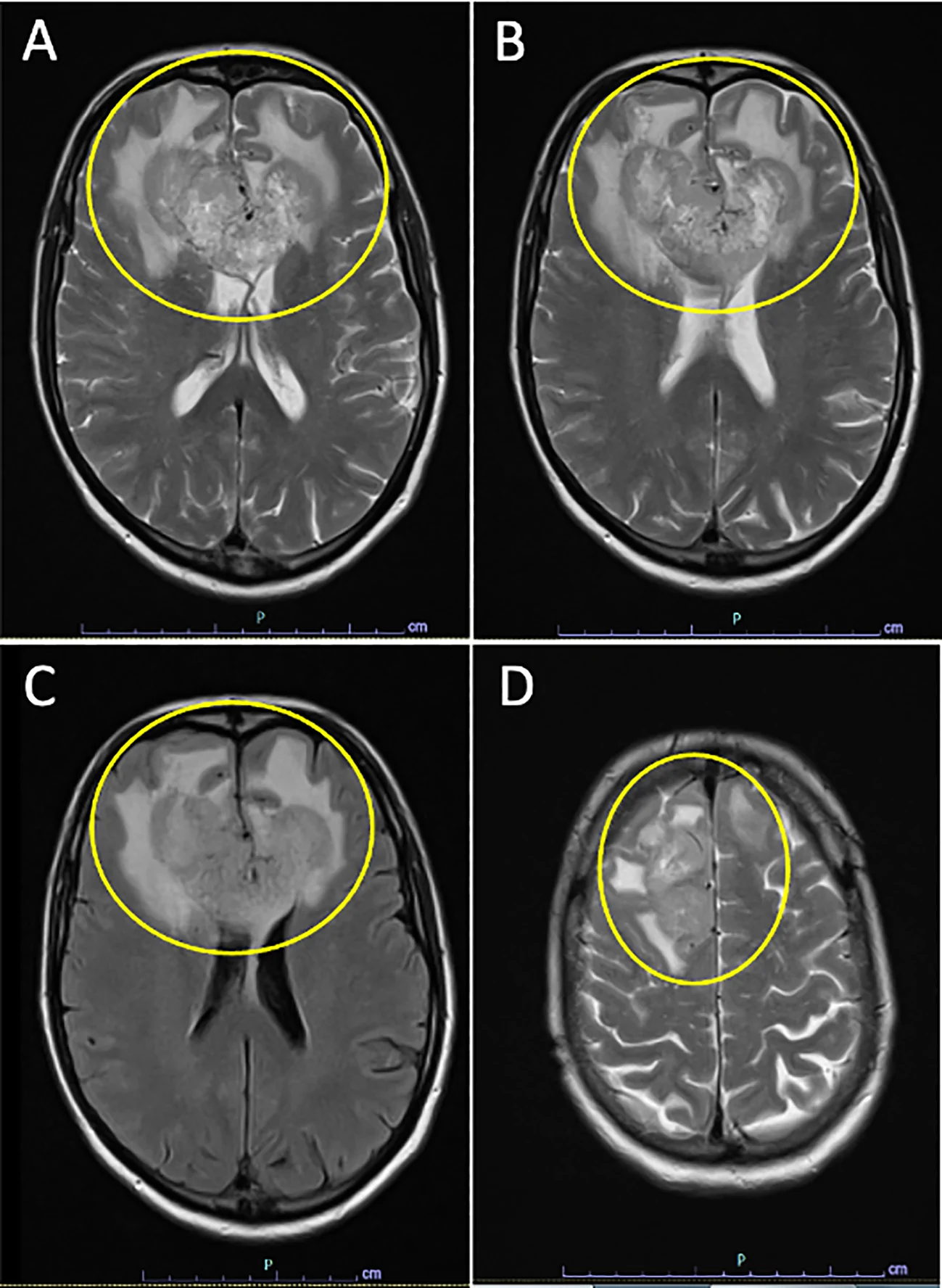
Cranial MRI **(A–D)** showing multifocal GBM with the main component centered at the genu of the corpus callosum and involving both frontal lobes, with an additional lesion in the right superior frontal gyrus. Yellow circles indicate the lesions.

The MRI had identified a probable multifocal GBM, with the main component centered at the genu of the corpus callosum involving both frontal lobes, and an additional component in the right superior frontal gyrus (**Figure 2**). Given the involvement of the corpus callosum and bilateral frontal extension, this case was classified as multifocal GBM with a callosal pattern of spread rather than multicentric GBM.

After exclusion of lesions outside the brain, and since the lesions were not amenable to surgical resection, he was proposed for a stereotactic brain biopsy. The immunohistochemical study from the biopsy showed GFAP positivity, ATRX preservation, p53 negativity, and non-mutated *IDH*, confirming the diagnosis of GBM.

Given the clinical deterioration, the patient was admitted to the Medical Oncology service for a course of dexamethasone as a therapeutic trial for suspected deterioration due to tumor-associated edema. Despite corticosteroid therapy, neurological and functional status did not improve. Serial assessments confirmed that the patient was not a candidate for oncologic therapy due to poor performance status. He was put on palliative care, and corticosteroids were gradually tapered.

The patient experienced progressive neurological deterioration and died approximately 2 months after diagnosis, without having received any tumor-directed therapy.

### 2.3 Case 3

**Figure 3:**
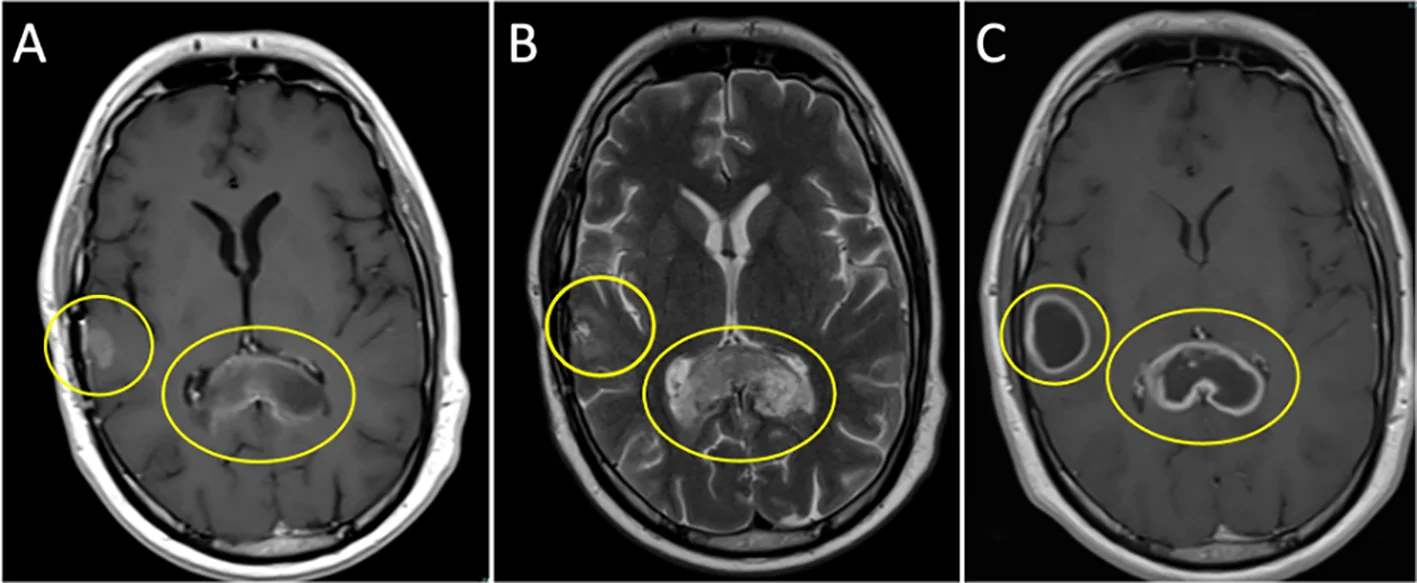
Cranial MRI **(A–C)** showing multifocal GBM with two supratentorial intra-axial lesions. One was located in the right temporo-opercular region, ovoid in shape, measuring 22.9 mm in latero-lateral diameter, 28.7 mm in anteroposterior (AP) diameter, and 25 mm in craniocaudal diameter. An additional bilateral hemispheric lesion is also present, with its epicenter in the splenium of the corpus callosum, showing moderate peripheral enhancement consistent with a cystic/necrotic lesion, measuring 48.4 mm in latero-lateral diameter, 23.7 mm in AP diameter, and 20.4 mm in craniocaudal diameter. Yellow circles indicate the right temporo-opercular lesion and the bilateral lesion centered in the splenium of the corpus callosum.

A 42-year-old man with no relevant past medical history presented with a first onset generalized tonic-clonic seizure accompanied by loss of consciousness. Initial cranial CT revealed two contrast-enhancing intra-axial lesions located in the right temporal lobe and the corpus callosum (forceps major), raising suspicion for high-grade malignancy. Subsequent contrast-enhanced MRI confirmed an infiltrative right temporo-opercular lesion with mild perilesional edema and a second bilateral lesion centered in the splenium of the corpus callosum, both showing peripheral enhancement, hemorrhagic components on susceptibility-weighted imaging, and perfusion features suggestive of malignancy (**Figure 3**). Whole-body positron emission tomography excluded extracranial tumor load. This case was classified as multifocal GBM because of the presence of two supratentorial lesions, including corpus callosum involvement, consistent with infiltrative spread along white matter pathways.

After discussion within the Neuro-Oncology multidisciplinary team meeting, the patient underwent neuronavigation-guided resection of the right temporal lesion. Histopathological analysis demonstrated a hypercellular infiltrative glial neoplasm with marked mitotic activity, necrosis, and microvascular proliferation. Immunohistochemistry showed positivity for GFAP, synaptophysin, and OLIG2, preserved ATRX expression, wild-type p53 pattern, and non-mutated *IDH1*, with a high proliferative index (Ki-67 approximately 60%), consistent with GBM. *MGMT* promoter methylation was detected.

Postoperatively, the patient recovered well without neurological deficits and maintained full functional independence (Eastern Cooperative Oncology Group (ECOG) performance status 0). He was started on antiepileptic therapy and corticosteroids with gradual tapering. Given his favorable performance status, he was proposed for standard concomitant chemoradiotherapy followed by adjuvant TMZ according to the Stupp protocol, which he initiated with good tolerance.

During treatment, the patient was clinically asymptomatic but remained dependent on corticosteroid therapy. To facilitate corticosteroid tapering and improve peritumoral edema control, off-label bevacizumab was indicated as a steroid-sparing strategy.

At the most recent follow-up, the patient was alive and receiving the adjuvant phase of the Stupp protocol, with an overall survival of 8 months from the date of histopathological diagnosis.

### 2.4 Case 4

**Figure 4:**
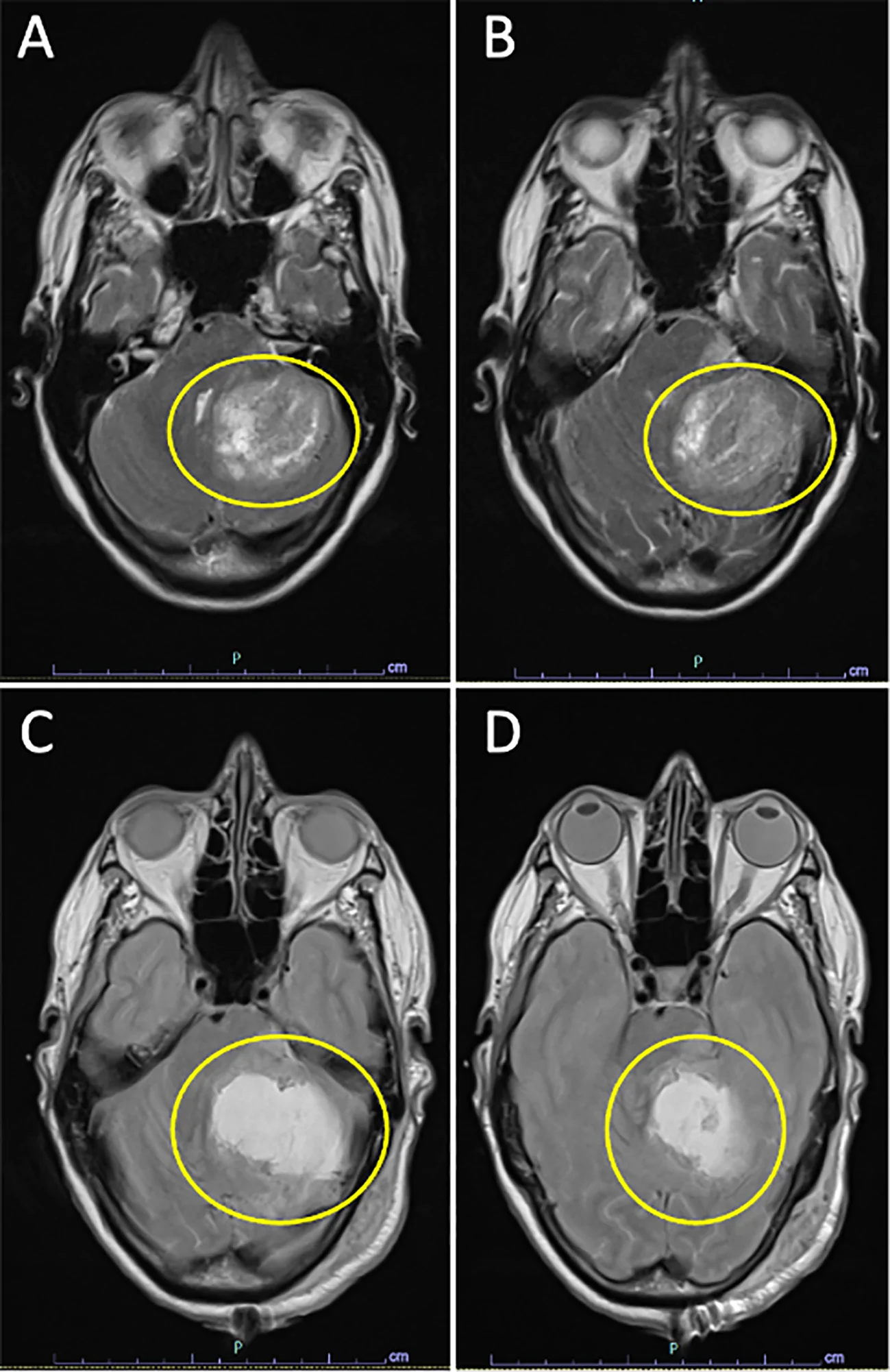
Cranial MRI **(A–D)** showing a left cerebellar intra-axial lesion (4 x 2.5 cm) with extension into the left cerebellar peduncle and brainstem. Yellow circles indicate the cerebellar lesion.

An 81-year-old woman presented with a three-day history of fatigue, left-sided hemiparesis and imbalance, dysarthria, and headaches. Her medical history was notable for arterial hypertension. Examination revealed marked dysarthria, left-sided dysmetria, and reduced left trigeminal sensation. Cranial CT revealed a left cerebellar mass causing brainstem compression and early hydrocephalus. MRI demonstrated a vascularized intra-axial lesion measuring 4 x 2.5 cm with cytotoxic edema and tumor infiltration extending into the left cerebellar peduncle and brainstem (**Figure 4**). She underwent suboccipital craniectomy, and the immunohistochemistry showed GFAP positivity, ATRX preservation, p53 negativity, and non-mutated *IDH*, consistent with the diagnosis of GBM.

On postoperative day three, she developed acute hydrocephalus secondary to worsening brainstem edema, requiring placement of an external ventricular drain, ventilatory support, and later tracheostomy. During intensive care, she developed critical illness myopathy but exhibited gradual neurological improvement, with partial recovery of consciousness and spontaneous limb movement. The follow-up MRI demonstrated enlargement of the brainstem signal abnormality, raising concern for tumor progression. Given the tumor location and clinical trajectory, there was no indication for adjuvant oncologic therapy, and palliative management was recommended. The external ventricular drain was removed during hospitalization, and further cerebrospinal fluid diversion was not pursued.

The patient remained on supportive care and died approximately 1.5 months after histopathological diagnosis.

## 3 Discussion

GBM remains the most common and aggressive primary malignant brain tumor in adults, with a peak incidence in the sixth to seventh decades of life, and an annual incidence of approximately 3.2 per 100,000 persons per year (1, 2, 17). Although most GBMs present as a single supratentorial lesion, multiple tumor foci, classified as either multifocal or multicentric, are increasingly recognized, accounting for 0.5-35% of cases depending on the diagnostic criteria and series examined (3, 4, 18). Multifocal GBM refers to multiple lesions connected by identifiable pathways, such as white matter tracts or cerebrospinal fluid dissemination, whereas multicentric GBM describes spatially discrete lesions without radiological or anatomical continuity, presumed to arise independently and representing an even rarer entity (4, 19). In routine clinical practice, however, this distinction is not consistently established, as it rarely influences therapeutic decision-making. Infratentorial GBM is exceptionally rare, accounting for approximately 0.4-3.4% of all GBM cases, and presents distinct diagnostic and therapeutic challenges related to its posterior fossa location (15, 16).

Applying these definitions to the present series, Cases 1 to 3 were classified as multifocal GBM, whereas no case was considered unequivocally multicentric. In Case 1, although the lesions were confined to the right hemisphere and appeared as multiple foci, the available imaging did not allow definitive exclusion of microscopic continuity. In Cases 2 and 3, the involvement of the corpus callosum supported a pattern of spread along commissural white matter pathways, favoring classification as multifocal rather than multicentric disease. Case 4 was classified separately as cerebellar/infratentorial GBM.

The four cases presented here illustrate the heterogeneity and clinical complexity associated with uncommon forms of GBM. Together, they underscore diagnostic ambiguity at initial presentation, limitations of available oncologic interventions in the context of advanced age, poor performance status, treatment-related toxicity or surgically constrained tumor location, and the poor prognosis associated with these variants.

The added value of reporting these four cases together lies in the comparative clinical perspective they provide. Although the patients differed in age, anatomical tumor distribution, functional status, treatment feasibility, and outcome (**Table 1**), their trajectories converged on several shared challenges that are insufficiently addressed in standard GBM management algorithms. First, uncommon GBM presentations may closely mimic other diagnostic entities, particularly metastatic disease in multifocal supratentorial presentations and other posterior fossa tumors in cerebellar disease. Second, the feasibility of standard treatment was determined less by histological diagnosis alone than by the interaction between tumor location, neurological status, surgical accessibility, molecular information, treatment tolerance, and the possibility of completing chemoradiotherapy. Third, these cases illustrate that multifocality does not uniformly preclude active treatment, as shown by the younger patient with preserved performance status, but it frequently reduces the likelihood of maximal resection and treatment completion. Together, these observations support the need for early individualized multidisciplinary assessment and highlight why shared clinical experience remains important for rare GBM presentations for which dedicated evidence-based recommendations are lacking.

**Table 1:** Summary of clinical, molecular, treatment, and outcome characteristics.

Case	Age/sex	Presentation	Tumor location	Molecular/histological features	Surgery/biopsy	Oncologic treatment	Overall Survival
1	70/M	Paresthesia, proximal weakness, weight loss	Five right-hemispheric supratentorial lesions	*IDH*-wildtype; ATRX preserved; p53 negative; GFAP positive; Ki-67 10–20%	Resection/biopsy of accessible right frontal lesion	Concomitant chemoradiotherapy; one adjuvant TMZ cycle	Death approximately 7.5 months after diagnosis
2	64/M	Confusion, memory impairment, incoherent speech, gait instability, sphincter dysfunction	Bifrontal lesion centered on genu of corpus callosum, additional right frontal lesion	*IDH*-wildtype; ATRX preserved; p53 negative; GFAP positive	Stereotactic biopsy	No tumor-directed therapy	Death 2 months after diagnosis
3	42/M	Generalized tonic-clonic seizure	Right temporo-opercular lesion and corpus callosum lesion	*IDH*-wildtype; ATRX preserved; p53 wild-type pattern; GFAP positive; Ki-67 ~60%; *MGMT* methylated	Resection of right temporal lesion	Stupp protocol	Alive at 8 months after diagnosis
4	81/F	Fatigue, hemiparesis, imbalance, dysarthria, headache	Left cerebellar mass with brainstem involvement and hydrocephalus	*IDH*-wildtype; ATRX preserved; p53 negative; GFAP positive	Suboccipital craniectomy	No tumor-directed therapy	Death 1.5 months after diagnosis

### 3.1 Diagnostic considerations and radiologic patterns

In our first case, the 70-year-old male patient presented with five right-hemispheric lesions, initially raising a strong suspicion for brain metastases, a common diagnostic pitfall in multifocal GBM. This presentation aligns with reports showing that multiple GBM frequently mimics metastatic disease radiologically, often requiring extensive extracranial investigation before the diagnosis is established (20, 21). Advanced MRI played a defining role in characterizing the lesions, and ultimately, a biopsy confirmed an *IDH*-wildtype GBM, consistent with the typical molecular profile of multifocal glial disease (1, 20).

Similarly, the second patient, a 64-year-old man, presented with a bifrontal lesion centered on the genu of the corpus callosum, a classic “butterfly glioma” configuration. Multifocal involvement through transcallosal spread is well described in GBM, reflecting infiltration via commissural white matter tracts (4, 19). As with Case 1, extensive systemic evaluation excluded a metastatic source (20).

A comparable multifocal pattern was observed in our third patient, a 42-year-old patient, who presented with two spatially distinct contrast-enhancing lesions involving the right temporal lobe and the splenium of the corpus callosum. This distribution, combining a lobar lesion with callosal involvement, further illustrates the propensity of GBM to spread along white matter tracts and across hemispheres. Histopathology again confirmed an *IDH*-wildtype GBM with high proliferative activity, reinforcing the biological consistency seen across multifocal GBM presentations. Notably, unlike the older patients, this individual maintained excellent functional status following partial resection and was able to proceed with standard concomitant chemoradiotherapy, highlighting the critical role of age and performance status in determining therapeutic feasibility rather than multifocality alone.

Collectively, these cases illustrate the broad clinical and biological continuum of multifocal and diffusely infiltrative gliomas. They emphasize that tumor distribution, involvement of commissural pathways such as the corpus callosum, and the resulting impact on neurological function are key determinants of prognosis and treatment eligibility, often surpassing histological classification alone.

In contrast, Case 4 describes an 81-year-old woman with a cerebellar GBM, an entity that represents a very small fraction of all GBMs. The clinical picture, characterized by ataxia, dysarthria, and early hydrocephalus, mirrors classic posterior fossa symptomatology reported in the literature (22). Although cerebellar GBM may radiologically resemble its supratentorial counterpart, its posterior fossa location imposes specific anatomical constraints, often limiting extensive resection and complicating postoperative management (16). In this patient, the rapid development of hydrocephalus requiring external ventricular drainage exemplifies the high morbidity associated with lesions compressing the brainstem and fourth ventricle.

The radiological differential diagnosis of an enhancing cerebellar mass in an elderly adult is broad and includes metastasis, hemangioblastoma, primary CNS lymphoma, infectious or inflammatory lesions such as abscess, and other high-grade gliomas. In the present case, the intra-axial infiltrative pattern, extension into the left cerebellar peduncle and brainstem, and associated mass effect with early hydrocephalus supported an aggressive primary glial tumor as an important diagnostic consideration. Nevertheless, definitive distinction from other posterior fossa lesions required histopathological confirmation, which established the diagnosis of GBM.

### 3.2 Therapeutic decision-making and barriers to standard treatment

The standard of care for GBM, maximal safe resection followed by combined chemoradiotherapy with TMZ (Stupp protocol), is associated with a median survival of 8–15 months in selected patients (2, 3, 5, 8). However, the applicability of this regimen is significantly restricted in multiple GBM and in infratentorial tumors due to anatomical, functional, and performance status limitations.

Safe maximal resection has relevant prognostic value, and multifocal cases preclude its achievement, with direct impact on survival and progression indexes. Also, multiple lesions are likely to increase neurological burden and worsen performance status, thereby compromising eligibility for adjuvant therapy. All of this is also true for posterior fossa location. In our series, gross total resection was not achievable. Cases 1 and 3 underwent resection of the most surgically accessible lesion, while Case 2 was limited to stereotactic biopsy. In Case 4, posterior fossa surgery was required, but brainstem proximity and postoperative deterioration restricted subsequent therapeutic options.

The first patient underwent biopsy followed by standard concurrent chemoradiotherapy and initiation of adjuvant TMZ. His clinical trajectory initially mirrored that of typical responders, but severe hematologic toxicity, grade 4 neutropenia, and grade 2 thrombocytopenia led to treatment interruption. Shortly after attempting to resume therapy, he experienced an abrupt neurological decline, a pattern frequently described in multifocal GBM where tumor burden and diffuse infiltration reduce the likelihood of sustained response even when therapy is technically feasible. His overall survival, approximately 7.5 months after diagnosis, falls within the 6–11-month range reported for multifocal GBM (3, 5, 6, 18), likely reflecting both treatment interruption and aggressive disease phenotype.

The second multifocal GBM patient exemplifies a different limitation: despite biopsy-confirmed GBM, he never became eligible for tumor-directed treatment due to progressive neurocognitive impairment and severely reduced performance status. Literature consistently identifies functional status as one of the strongest prognostic factors in both single and multiple GBM, and patients with multifocal disease often present with more profound deficits, precluding resection or intensive oncologic therapy (3, 18). His overall survival of approximately 2 months after diagnosis is consistent with the worst prognosis of the multifocal GBM series, in which survival is often limited to weeks when tumor-directed therapy cannot be administered.

In the case of the 42-year-old patient, despite the presence of multifocal disease involving the right temporal lobe and corpus callosum, several favorable factors supported the use of standard oncologic therapy. These included young age, preserved neurological function, excellent postoperative recovery, and an ECOG performance status of 0. Partial surgical resection of the dominant temporal lesion allowed histological confirmation of *IDH*-wildtype GBM and symptomatic control, enabling subsequent initiation of concomitant chemoradiotherapy followed by adjuvant TMZ according to the Stupp protocol. This case exemplifies that multifocality alone does not necessarily preclude standard treatment when functional status is preserved and at least one lesion is surgically accessible. Similar observations have been reported in the literature, where selected patients with multifocal GBM derive survival benefit from aggressive multimodal therapy when performance status allows it (23).

Our observations are consistent with published reports showing that completion of the full Stupp protocol is less frequent in patients with multifocal or anatomically constrained GBM than in selected patients with unifocal supratentorial disease. In the present series, only the youngest patient with preserved ECOG performance status was able to initiate standard concomitant chemoradiotherapy, although TMZ still required dose reduction and discontinuation because of hematologic toxicity. By contrast, one patient could not receive tumor-directed treatment because of poor performance status, one discontinued treatment after severe hematologic toxicity and neurological decline, and the cerebellar GBM patient was not eligible for adjuvant therapy after postoperative deterioration.

These observations reinforce that eligibility for the standard Stupp regimen is primarily dictated by performance status, neurological stability, treatment tolerance, and the possibility of safe surgical intervention rather than multifocality alone. While younger age and favorable molecular markers are generally associated with improved outcomes, they do not guarantee access to aggressive treatment in the setting of extensive, functionally devastating tumor infiltration. Early multidisciplinary assessment is therefore essential to balance potential oncologic benefit against treatment-related morbidity and to integrate supportive and palliative care when standard therapy is not feasible.

In cerebellar GBM, maximal safe resection remains the cornerstone of management, yet true gross-total removal is rarely achievable due to proximity to the brainstem and deep cerebellar nuclei (15, 16). In Case 4, the patient underwent suboccipital craniectomy, but postoperative hydrocephalus and critical illness myopathy significantly impaired recovery. Given the tumor location, the radiologic suggestion of early progression, and the advanced age, the patient was not considered a candidate for adjuvant chemoradiotherapy. The disease course, death approximately one month after admission, reflects the dismal survival associated with infratentorial GBM, which often presents emergently, progresses rapidly, and lacks robust therapeutic options (16).

When analyzed together, the four cases show that rare GBM presentations are clinically heterogeneous but share several management challenges. Cases 1 to 3 presented as multifocal supratentorial GBM and initially raised important diagnostic concerns, particularly the need to exclude metastatic disease. However, their therapeutic trajectories differed markedly. Case 3 illustrates that multifocality alone does not preclude standard treatment when age, performance status, and surgical accessibility are favorable. By contrast, Cases 1 and 2 demonstrate how treatment completion may be limited by toxicity or poor neurological status. Case 4, although anatomically distinct as a cerebellar GBM, reinforces the same central principle: anatomical constraints and early neurological deterioration can rapidly eliminate the possibility of adjuvant oncologic therapy. Thus, across all cases, performance status, lesion location, surgical accessibility, and treatment tolerance emerged as decisive determinants of management and outcome.

### 3.3 Prognosis and survival in the context of multifocality and rare location

Although classification of GBM as multifocal or multicentric provides valuable insights into tumor dissemination patterns, several studies suggest that the distinction has limited prognostic relevance when compared to determinants of outcome, such as patient age, *MGMT* promoter methylation status (patients with *MGMT* promoter methylation respond better to TMZ), functional performance, and feasibility of the treatment. Nevertheless, multiple GBMs generally demonstrate poorer prognosis than single tumors, with shorter median survival and reduced likelihood of surgical cytoreduction (3, 18, 24). Our cases align with the literature: two patients had significantly abbreviated survival, reflecting both disease burden and barriers to aggressive treatment. Although *MGMT* promoter methylation is an important predictor of responsiveness to TMZ, *MGMT* status was assessed in only one patient in our series and was found to be methylated; notably, this patient had the most favorable clinical course. In the remaining cases, severely impaired functional status would likely have precluded chemotherapy irrespective of *MGMT* status.

Although infratentorial GBM is biologically comparable to its supratentorial counterpart, it presents additional diagnostic and therapeutic challenges owing to its critical anatomical location. As observed in Case 4, the combination of mass effect on the brainstem, rapid development of hydrocephalus, and postoperative complications limited therapeutic options and contributed to a poor prognosis.

In this context, the outcomes observed in our series were at the unfavorable end of the spectrum. Case 1 survived approximately 7.5 months after diagnosis, Case 2 died approximately 2 months after diagnosis, and Case 4 died approximately 1.5 months after diagnosis. These abbreviated outcomes are likely explained by the combination of extensive tumor burden, advanced age or neurological decline, inability to achieve extensive cytoreduction, postoperative complications, and incomplete or absent delivery of tumor-directed therapy. Conversely, Case 3 had the most favorable early course, supporting the concept that selected patients with multifocal GBM may still benefit from multimodal therapy when performance status, surgical accessibility, and molecular features are favorable.

All cases highlight the critical role of supportive and palliative care in GBM management, particularly when standard oncologic therapies are not feasible. Corticosteroids remain indispensable for reducing peritumoral edema, especially in multifocal disease where mass effect arises from multiple foci. Seizure management and symptom control are central to maintaining quality of life. In our series, two patients transitioned early to palliative-focused care when the probability of meaningful clinical improvement with oncologic therapy was deemed minimal, consistent with expert recommendations for patients with extensive multifocal involvement, severe functional decline, or high-risk posterior fossa tumors (9–11).

### 3.4 Implications and future directions

The presented cases highlight persistent gaps in the understanding and management of rare GBM variants. Multifocal disease often mimics metastatic tumors, necessitating extensive diagnostic evaluation, while infratentorial lesions may resemble other posterior fossa pathologies, complicating early recognition. Therapeutically, both multifocal and cerebellar GBMs frequently limit the feasibility of maximal resection or combined chemoradiotherapy, resulting in poorer outcomes and markedly shortened survival, especially when functional status is compromised. Current treatment strategies remain largely adapted from single-lesion GBM, as no dedicated, evidence-based guidelines exist for these uncommon presentations. Continued research is needed to refine diagnostic tools and develop tailored approaches for patients with these challenging forms of GBM.

### 3.5 Methodological considerations and limitations

Several methodological considerations should be acknowledged when interpreting this case series. First, it is a retrospective single-center case series including only four patients, which limits the generalizability of the findings and precludes any formal statistical analysis. Nevertheless, given the rarity of multifocal and cerebellar GBM, detailed real-world descriptions remain clinically valuable, particularly when they illustrate diagnostic uncertainty, treatment-limiting factors, and multidisciplinary decision-making in uncommon presentations for which dedicated evidence-based recommendations are lacking.

Second, the cases were clinically heterogeneous regarding age, anatomical tumor distribution, performance status, surgical approach, treatment feasibility, and outcome. This heterogeneity limits direct comparison between patients but also reflects the clinical reality of rare GBM presentations. In this context, the aim of the present series was not to define an optimal therapeutic strategy, but rather to highlight recurring clinical challenges, including the difficulty of distinguishing multifocal GBM from metastatic disease, the limited feasibility of maximal safe resection, the frequent inability to complete standard chemoradiotherapy, and the importance of early individualized multidisciplinary assessment.

Third, molecular characterization was incomplete in some cases. Although all tumors were consistent with *IDH*-wildtype GBM, additional molecular markers recommended in the context of the 2021 WHO classification were not systematically available. *MGMT* promoter methylation status was available in only one patient (Case 3), in which promoter methylation was detected, whereas *TERT* promoter mutation, *EGFR* amplification, and chromosome +7/-10 status were not performed or were unavailable in the remaining cases. This limits the biological interpretation of the series and restricts correlations between molecular profile, treatment response, and survival. Nevertheless, this reflects the limitations frequently encountered in real-world clinical practice, particularly in rapidly progressive cases, patients with poor performance status, or situations in which limited biopsy material is available.

Fourth, treatment exposure and follow-up differed substantially among patients. Some patients were unable to initiate tumor-directed therapy, whereas others started but could not complete standard treatment because of toxicity or clinical deterioration. Therefore, survival differences across cases should be interpreted cautiously, as they likely reflect the combined effects of tumor biology, anatomical location, neurological status, surgical accessibility, treatment tolerance, and supportive care decisions. For these reasons, the present case series should be regarded as an educational and hypothesis-generating contribution rather than as evidence supporting a standardized management approach.

Finally, because rare GBM variants remain underrepresented in prospective studies, larger multicenter registries and collaborative studies are needed to better define their molecular features, natural history, treatment completion rates, prognostic factors, and optimal management strategies. This is particularly relevant for multifocal and infratentorial GBM, where current management is still largely extrapolated from evidence generated in patients with unifocal supratentorial disease.

## 4 Conclusions

The four cases presented illustrate the clinical heterogeneity and therapeutic complexity of rare GBM presentations, including multifocal supratentorial disease and infratentorial cerebellar GBM. Although their radiologic and anatomical features differed substantially, all cases shared a uniformly poor prognosis, reflecting well-established challenges in the management of non-unifocal and surgically constrained GBM. Multifocal GBM frequently mimics metastatic disease at presentation and often precludes maximal resection or eligibility for the full Stupp protocol, while cerebellar GBM poses unique risks due to its proximity to the brainstem and potential for life-threatening hydrocephalus.

In most cases, definitive treatment was limited either by advanced age, functional decline, hematologic toxicity, or anatomical considerations, ultimately leading to abbreviated survival despite timely diagnosis. These clinical trajectories underscore that, beyond molecular classification, functional status and the feasibility of tumor-directed therapy remain the most powerful determinants of outcome in patients with rare glioblastoma variants.

Given the absence of dedicated evidence-based guidelines for multifocal or infratentorial GBM, the management of these patients continues to rely on extrapolation from unifocal supratentorial GBM and on individualized multidisciplinary decision-making. Clinically, our cases reinforce the importance of early multidisciplinary assessment to define realistic treatment goals, identify patients who may still benefit from multimodal therapy, and integrate supportive or palliative care when standard treatment is unlikely to provide meaningful benefit. Larger prospective and multicenter studies are needed to better define the biology, prognostic factors, treatment completion rates, and optimal management strategies for these challenging and uncommon GBM presentations.

## Data Availability

The original contributions presented in the study are included in the article/supplementary material. Further inquiries can be directed to the corresponding author.

## References

[1] Louis DN Perry A Wesseling P Brat DJ Cree IA Figarella-Branger D . The 2021 WHO classification of tumors of the central nervous system: a summary. Neuro Oncol. (2021) 23:1231–51. doi: 10.1093/neuonc/noab106 34185076 PMC8328013

[2] Price M Ballard CAP Benedetti JR Kruchko C Barnholtz-Sloan JS Ostrom QT . CBTRUS statistical report: Primary brain and other central nervous system tumors diagnosed in the United States in 2018-2022. Neuro Oncol. (2025) 27:iv1–66. doi: 10.1093/neuonc/noaf194 41092086 PMC12527013

[3] Haque W Thong Y Verma V Rostomily R Brian Butler E Teh BS . Patterns of management and outcomes of unifocal versus multifocal glioblastoma. J Clin Neurosci. (2020) 74:155–9. doi: 10.1016/j.jocn.2020.01.086 32089384

[4] Thomas RP Xu LW Lober RM Li G Nagpal S . The incidence and significance of multiple lesions in glioblastoma. J Neuro-Oncol. (2013) 112:91–7. doi: 10.1007/s11060-012-1030-1 23354652

[5] Patil CG Yi A Elramsisy A Hu J Mukherjee D Irvin DK . Prognosis of patients with multifocal glioblastoma: a case-control study. J Neurosurg. (2012) 117:705–11. doi: 10.3171/2012.7.JNS12147 22920963

[6] Paulsson AK Holmes JA Peiffer AM Miller LD Liu W Xu J . Comparison of clinical outcomes and genomic characteristics of single focus and multifocal glioblastoma. J Neuro-Oncol. (2014) 119:429–35. doi: 10.1007/s11060-014-1515-1 24990827 PMC4146694

[7] Grochans S Cybulska AM Simińska D Korbecki J Kojder K Chlubek D . Epidemiology of glioblastoma multiforme-literature review. Cancers (Basel). (2022) 14:2412. doi: 10.3390/cancers14102412 35626018 PMC9139611

[8] Stupp R Mason WP van den Bent MJ Weller M Fisher B Taphoorn MJB . Radiotherapy plus concomitant and adjuvant temozolomide for glioblastoma. N Engl J Med. (2005) 352:987–96. doi: 10.1056/NEJMoa043330 15758009

[9] Weller M van den Bent M Preusser M Le Rhun E Tonn JC Minniti G . EANO guidelines on the diagnosis and treatment of diffuse gliomas of adulthood. Nat Rev Clin Oncol. (2021) 18:170–86. doi: 10.1038/s41571-020-00447-z 33293629 PMC7904519

[10] Arakawa Y Mineharu Y Uto M Mizowaki T . Optimal managements of elderly patients with glioblastoma. Jpn J Clin Oncol. (2022) 52:833–42. doi: 10.1093/jjco/hyac075 35552425 PMC9841411

[11] Mazarakis NK Robinson SD Sinha P Koutsarnakis C Komaitis S Stranjalis G . Management of glioblastoma in elderly patients: a review of the literature. Clin Trans Radiat Oncol. (2024) 46:100761. doi: 10.1016/j.ctro.2024.100761 38500668 PMC10945210

[12] Lasocki A Gaillard F Tacey M Drummond K Stuckey S . Multifocal and multicentric glioblastoma: improved characterisation with FLAIR imaging and prognostic implications. J Clin Neurosci. (2016) 31:92–8. doi: 10.1016/j.jocn.2016.02.022 27343042

[13] Ma R Taphoorn MJB Plaha P . Advances in the management of glioblastoma. J Neurol Neurosurg Psychiatry. (2021) 92:1103–11. doi: 10.1136/jnnp-2020-325334 34162730

[14] Farhat M Fuller GN Wintermark M Chung C Kumar VA Chen M . Multifocal and multicentric glioblastoma: imaging signature, molecular characterization, patterns of spread, and treatment. Neuroradiol J. (2023) 19714009231193162. doi: 10.1177/19714009231193162 37559514

[15] Lizunou Y Potthoff AL Schäfer N Waha A Borger V Herrlinger U . Cerebellar glioblastoma in adults: a comparative single-center matched pair analysis and systematic review of the literature. J Cancer Res Clin Oncol. (2024) 150:432. doi: 10.1007/s00432-024-05959-0 39340649 PMC11438707

[16] Zhang M Li R Pollom EL Amini A Dandapani S Li G . Treatment patterns and outcomes for cerebellar glioblastoma in the concomitant chemoradiation era: a National Cancer database study. J Clin Neurosci. (2020) 82:122–7. doi: 10.1016/j.jocn.2020.10.049 33317719 PMC7738760

[17] Tamimi AF Juweid M . Epidemiology and outcome of glioblastoma. In: De Vleeschouwer S , editor. Glioblastoma. Codon Publications, Brisbane (AU (2017). Available online at: http://www.ncbi.nlm.nih.gov/books/NBK470003/ (Accessed April 6, 2026). 29251870

[18] Baro V Cerretti G Todoverto M Della Puppa A Chioffi F Volpin F . Newly diagnosed multifocal GBM: a monocentric experience and literature review. Curr Oncol. (2022) 29:5. doi: 10.3390/curroncol29050280 35621670 PMC9139839

[19] Batzdorf U Malamud N . The problem of multicentric gliomas. J Neurosurg. (1963) 20:122–36. doi: 10.3171/jns.1963.20.2.0122 14192080

[20] Müller SJ Khadhraoui E Ernst M Rohde V Schatlo B Malinova V . Differentiation of multiple brain metastases and glioblastoma with multiple foci using MRI criteria. BMC Med Imaging. (2024) 24:3. doi: 10.1186/s12880-023-01183-3 38166651 PMC10759655

[21] Zhang ZX Chen JX Shi BZ Li GH Li Y Xiang Y . Multifocal glioblastoma—two case reports and literature review. Chin Neurosurg Jl. (2021) 7:8. doi: 10.1186/s41016-020-00223-z 33446281 PMC7809824

[22] Kikuchi K Hiratsuka Y Kohno S Ohue S Miki H Mochizuki T . Radiological features of cerebellar glioblastoma. J Neuroradiol. (2016) 43:260–5. doi: 10.1016/j.neurad.2015.10.006 26740386

[23] Patil CG Yi A Elramsisy A Hu J Mukherjee D Irvin DK . Prognosis of patients with multifocal glioblastoma: a case-control study. J Neurosurg. (2012) 117:705–11. doi: 10.3171/2012.7.JNS12147 22920963

[24] Guerrini F Mazzeo LA Rossi G Verlotta M Del Maestro M Rampini AD . Is it worth considering multicentric high-grade glioma a surgical disease? Analysis of our clinical experience and literature review. Tomography. (2021) 7:523–32. doi: 10.3390/tomography7040045 34698304 PMC8544720

